# A Systematic Review and Meta-Analysis of Short-Term Ambient Ozone Exposure and COPD Hospitalizations

**DOI:** 10.3390/ijerph17062130

**Published:** 2020-03-23

**Authors:** Hui Gao, Kan Wang, William W. Au, Wensui Zhao, Zhao-lin Xia

**Affiliations:** 1 Changning Center for Disease Control and Prevention, Shanghai 200051, China; gaohui@cncdc.org; 2 School of Public Health, & Key Laboratory of Public Health Safety of Ministry of Education of China, Fudan University, Shanghai 200032, China; k.wang@erasmusmc.nl; 3 Department of Epidemiology, Erasmus Medical Center, 3000CA Rotterdam, The Netherlands; 4 University of Medicine, Pharmacy, Science and Techonology, 540142 Tirgu Mures, Romania; wau@stu.edu.cn; 5 Faculty of Preventive Medicine and MPH Education Center, Shantou University Medical College, Shantou 515041, China

**Keywords:** ozone, chronic obstructive pulmonary disease, environmental health, meta-analysis

## Abstract

Chronic obstructive pulmonary disease (COPD) is the third leading cause of death globally and ozone exposure is a main cause of its disease burden. However, studies on COPD hospitalizations from short-term ambient level ozone exposure have not generated consensus results. To address the knowledge gap, comprehensive and systematic searches in several databases were conducted using specific keywords for publications up to February 14, 2020. Random-effect models were used to derive overall excess risk estimates between short-term ambient-level ozone exposure and COPD hospitalizations. The influence analyses were used to test the robustness of the results. Both meta-regression and subgroup analyses were used to explore the sources of heterogeneity and potential modifying factors. Based on the results from 26 eligible studies, the random-effect model analyses show that a 10 µg/m^3^ increase in maximum 8-h ozone concentration was associated with 0.84% (95% CI: 0.09%, 1.59%) higher COPD hospitalizations. The estimates were higher for warm season and multiple-day lag but lower for old populations. Results from subgroup analyses also indicate a multiple-day lag trend and bigger significant health effects during longer day intervals. Although characteristics of individual studies added modest heterogeneity to the overall estimates, the results remained robust during further analyses and exhibited no evidence of publication bias. Our systematic review and meta-analysis indicate that short-term ambient level ozone exposure was associated with increased risk of COPD hospitalizations. The significant association with multiple-day lag trend indicates that a multiple-day exposure metric should be considered for establishing ambient ozone quality and exposure standards for improvement of population health. Future investigations and meta-analysis studies should include clinical studies as well as more careful lag selection protocol.

## 1. Introduction

Chronic obstructive pulmonary disease (COPD), which is characterized by progressive and irreversible airflow limitation and chronic inflammation of the lungs, is the third leading cause of death globally [1]. According to the Global Burden of Disease (GBD) Study 2015 [2], exposures to ambient particulate matter and ozone have been ranked as the second and fifth biggest causes of COPD, respectively. Although the relationship between ambient particulate matter and COPD has been widely investigated [3,4,5,6], that for ozone is less extensive and the results are less consistent [7,8]. In the 2006 Air Quality Criteria for Ozone report [9], the association between short-term ozone exposure and respiratory effects has been emphasized, but this is based mainly on epidemiological studies regarding total respiratory diseases and/or asthma, not specifically on COPD. Since then, studies based on ozone exposure and hospital admissions have been increasingly investigated. For example, several valuable studies which were conducted in Iran [10], Italy [11], France [12] have also put further insight into this field.

Meta-analysis is an important approach to synthesizing evidence across studies. So far, four analyses have been conducted to quantify the association between short-term ambient ozone exposure and COPD hospitalizations [13,14,15,16] (Table S1): one was focused solely on East Asia [13], one was based on publications before 2008 [14], and the other two, conducted in 2015 [15] and 2016 [16], simply reported pooled risk estimates. Since then, several original investigations have been published [17,18,19,20]. In addition, a major issue has been raised regarding exposure to other air pollutants simultaneously, which may confound ozone-related effect estimates [21]. Indeed, there has not been another meta-analysis to summarize the more recent publications and address impacts from exposure to co-pollutants. The lack of an updated meta-analysis and pooled risk estimates would limit the ability to fully understand the impact of ambient level ozone exposure on COPD development.

To provide an updated and better understanding of the association between short-term ambient ozone exposure and COPD hospitalizations, a systematic review and meta-analysis were conducted with strict inclusion/exclusion criteria and with attention to the source of effect variations.

## 2. Material and Methods

### 2.1. Search Strategy

With no start date specified, four literature databases (PubMed, Web of Science, Medline and Embase) were searched for publications in English before September 19, 2019. The search was further updated in February 14, 2020. Keywords used included “chronic obstructive pulmonary disease,” “COPD,” “ozone,” and “O_3_”, alone and in combination. After excluding duplications using NoteExpress, the titles and abstracts from the publications were reviewed. The potentially eligible ones were selected and retrieved as full texts. Furthermore, references in previous meta-analysis papers were screened for any pertinent articles that were not previously identified. Finally, the acquired articles were reviewed and filtered according to the inclusion and exclusion criteria.

**Figure 1:**
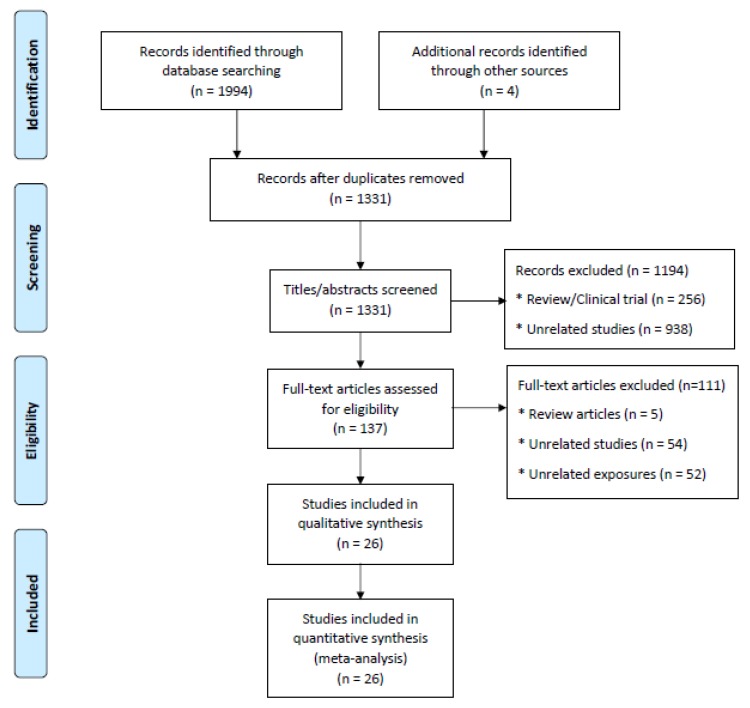
Study selection flowchart (searching before September 19, 2019).

Selection of publications was undertaken independently by two of the authors (H.G. and K.W.). Afterwards, the two authors discussed their results and disagreements were settled by consensus. A flow diagram for details of the search process in the meta-analysis is given in Figure 1.

### 2.2. Inclusion Criteria

The criteria were: (1) original studies; (2) study design: case crossover or time series; (3) reliable exposure measurement: ozone exposure was estimated by research grade methods or national monitoring station, reported with no missing or only a few missing days; (4) COPD definition: the International Classification of Disease (ICD), Ninth or Tenth Revision; (5) clear outcome measurements: studies reported quantitative measures of association, or gave sufficient data to calculate the excess risk (ER) and 95% CI; (6) results evaluated the influence of short-term exposure to ambient ozone.

### 2.3. Exclusion Criteria

The criteria were: (1) incomplete information; (2) non-observational study design; (3) non-original studies; (4) did not provide calculable or reported ER/relative risk (RR)/odds ratio (OR)/percent change and 95% CI; (5) uncertain diagnosis of COPD, or diagnosis according to pulmonary function test; (6) patients with COPD and asthma, or did not mention whether asthma was excluded.

### 2.4. Data Extraction

The following data from the eligible publications were extracted: first author, year of publication, study location, study design, study period, assessment of ambient ozone exposure, characteristics of temperature, detailed definition of outcome, data source (emergency department visits/hospital admissions), pollutant model, confounding factors, and statistical model. Data on effect estimates (single-pollutant and multi-pollutant models) of the association (and their 95% CI) between ambient ozone exposure and COPD hospitalizations were also extracted.

The data extraction process was also undertaken independently by two of the authors (H.G. and K.W.) and disagreements were settled by consensus. The characteristics of the included studies [17,18,19,20,22,23,24,25,26,27,28,29,30,31,32,33,34,35,36,37,38,39,40,41,42,43] are shown in Tables S1, S2, and S3.

### 2.5. Risk-of-Bias Assessment

Although researchers have suggested to evaluate the quality of time series or case crossover studies [44], there were still no validated scales recommended [45]. Unlike previous meta-analyses [15,16] which just assessed the risk-of-bias or quality subjectively, based on exposure assessment, outcome definition, adjustments of cofounders, etc., our study involved an initial search based on more strict inclusion/exclusion criteria (see above). This also explains why the number of included publications in our study is relatively smaller than the previous meta-analyses. After excluding unqualified studies, the quantitative meta-analysis in our study was performed using data collected from homogeneous backgrounds (see below). Thus, the specific risk-of-bias assessment services were not needed here.

### 2.6. Data Synthesis

Excess risk (ER, %) was pooled from appropriate studies per 10 µg/m^3^ increment in ambient maximum 8-h ozone concentration. This metric has been reported as closely associated with respiratory health [46]. However, for the study that reported results using a different exposure metric, other methods were used: estimates were based on maximum 8-h average ozone concentration or converted from other metrics by a standard ratio, a relationship of 20: 15: 8 for 1-h maximum: maximum 8-h average: 24-h average [47]. Our assumption was that 1 ppb equals 1.96 µg/m^3^, which would ensure comparable units. The OR or RR was also converted to ER in accordance with a previous study [48]. Briefly, considering that the increased risk of COPD hospitalizations would be very low, the measurements of OR and RR would be almost equivalent. Therefore, a transformation formula was used:

(1) $$\mathrm{ER}=\left(e^{\frac{\ln\left(RR^{*}\right)}{IQR}\times 10}-1\right)\times 100\%$$

where $RR^{*}$ and *IQR* were the relative risk and exposure metrics used in the original study.

Some studies reported multiple estimates as several lagged days, age groups, seasons, or other factors. Considering the bias that age [40], temperature [47], co-pollutants [49], or lag [14] might have caused, it was necessary to include only one estimate from each study with the shortest lag for all ages, all years, and without adjustment for co-pollutants for the calculation of overall excess risk estimates, except when a single study provided results from different temperature levels or multicity results. Each temperature-specific or city-specific result would be included. The same chosen standard was also used to pool the level-specific estimate during the subgroup analyses, except for the level explored in our study.

### 2.7. Statistical Methods

Due to different study designs, methods of analysis, different lag exposures, geographical and population differences, and experiences from previous studies [14,44,50], heterogeneity between studies was anticipated and a random-effect model was used to account for both within- and between- study heterogeneity. The standard *I*^2^ test was used to examine the heterogeneity. A funnel plot and an Egger test were used to investigate publication bias.

Influence analyses were performed using both covariance ratio and hat plots to identify possible influential cases [51]. After excluding influential cases, “leave-one-out” analyses were performed to test the robustness of our results. Meta-regression was used to investigate the possible modification effects of publication year, study location, study design, data source, definition of outcome and influenza-adjusted status on the overall effect sizes of ozone, which would provide more information on the source of the heterogeneity among the included studies. Furthermore, subgroup analyses were performed to investigate how estimates differed by age, temperature, lag structure and co-pollutants.

All statistical analyses were performed with the R software, version 3.6.1 (R Foundation for Statistical Computing, Vienna, Austria), using the “meta” “metafor” “dmetar” and “forestplot” packages. A *p* value of less than 0.05 was considered to indicate statistical significance.

This meta-analysis was conducted in accordance with Preferred Reporting Items for Systematic review and Meta-Analyses (PRISMA) guidelines [52].

## 3. Results

### 3.1. Search Findings and Study Characteristics

The initial literature searches yielded 1998 documents (PubMed: 231; Web of Science: 241; Medline: 1073; Embase: 449; Other Source: 4), of which 1976 were excluded based on duplication, title and abstract, and full text review. Therefore, 26 original articles were selected for further analyses.

Table 1 provides an overview of all the included studies, 19 were time series studies, while 7 were case crossover studies. Most studies were conducted in North America, Europe, and China. Nearly half of the eligible studies adjusted for influenza effects in their reported results. Most studies used the ICD-9 COPD definition. Additionally, Table S2 provides detailed information about study period, COPD definition and the characteristics of ambient ozone level and temperature for the included studies, while Table S3 provides a summary of the adjusted confounders and statistical models used.

### 3.2. Ambient Level Ozone Exposure and COPD Hospitalizations

**Figure 2:**
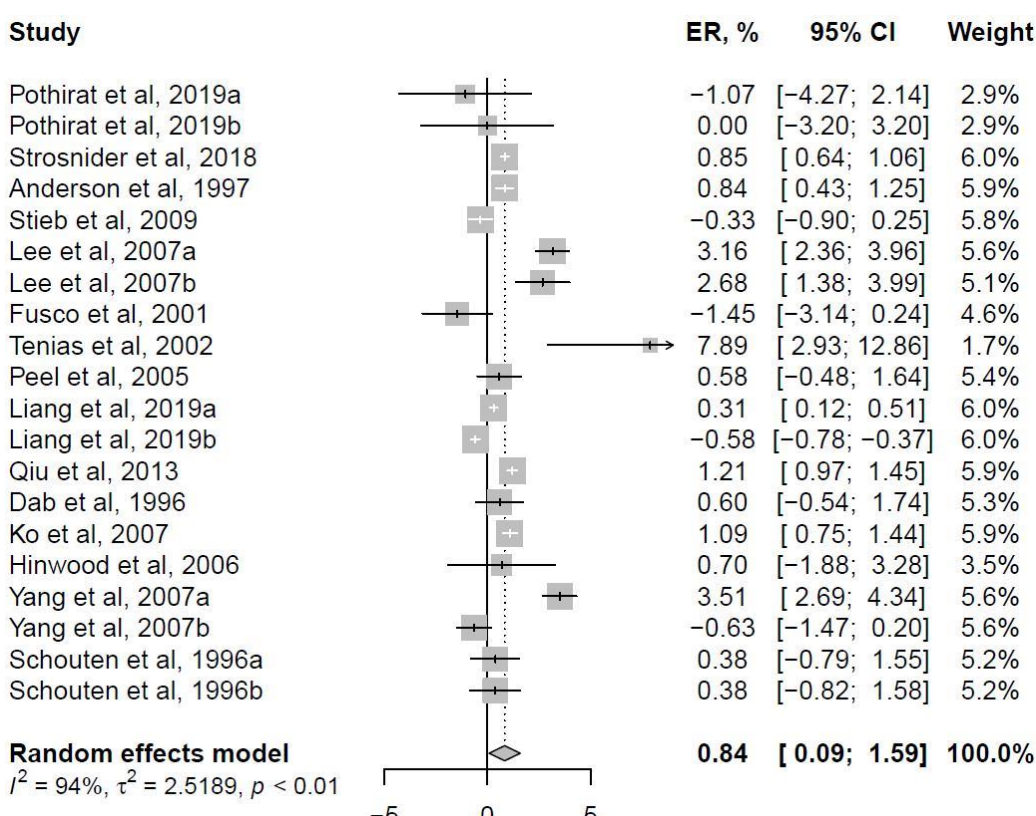
Forest plot of short-term ambient level ozone exposure and COPD hospitalizations. Excess risk (ER) of COPD hospitalizations per 10 µg/m^3^ increase in maximum 8-h ozone concentration.

A total of 15 studies with 20 estimates were used to pool overall ER between short-term ambient level ozone exposure and COPD hospitalizations in all age-range and year-round exposure. As illustrated in Figure 2, results from the random-effect model show that a 10 µg/m^3^ increase in maximum 8-h ozone concentration was associated with 0.84% (95% CI: 0.09%, 1.59%) higher COPD hospitalizations.

Additionally, influence analyses (Figure S1) were used to test the robustness of the overall estimates. After removing the potentially influential case [27], the overall ER estimate was 0.74% (95% CI: 0.11%, 1.37%), which remained significant with a narrow interval (Figure S2). Meanwhile, the leave-one-out analyses (Figure S3) also show consistent results, with relatively smaller but significant effect sizes.

### 3.3. Publication Bias

The Egger test and funnel plot were used to investigate publication bias. The funnel plot analyses did not reveal asymmetry results (Figure S4). The estimates based on the Egger test (bias = 1.32, *p* = 0.336) further suggest that publication bias did not exist in the overall effect estimate. When the influential case was excluded, the results from the funnel plot and the Egger test (Figure S5. bias = 1.12, *p* = 0.444) remained robust.

### 3.4. Heterogeneity by Meta-Analysis

Meta-regression analyses were used to investigate potential sources of heterogeneity, according to a set of key study characteristics including publication year, study design, study location, definition of outcome, data source and influenza. The results indicate that all these factors contributed to the heterogeneity (data not shown). The results from the study design show modifying effects at the borderline significant level (Figure S6. *β* = −1.52, *p* = 0.061), which gained significance after the influential case was removed (Figure S7. *β* = −1.67, *p* = 0.010).

### 3.5. Subgroup Analyses

Subgroup analyses were conducted to explore the effects of age, season, co-pollutants and lag structure. To untangle complicated effects among different factors, only estimates from all age-range and year-round exposure were used to calculate the pooled results for co-pollutants and different lag structures. In addition, year-round exposure data were used to investigate the effect of age and all age ranges, to explore seasonal variations.

**Figure 3:**
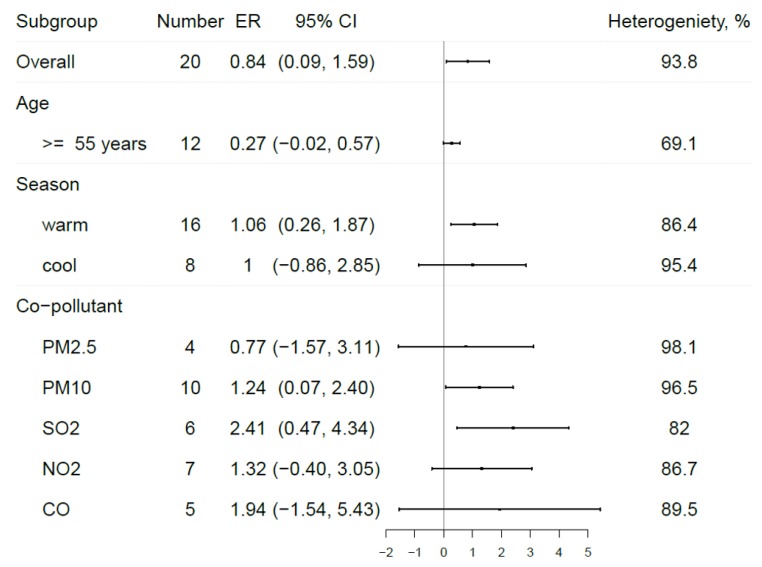
Subgroup analyses among different age ranges, seasons and co-pollutants. Note: ER, excess risk.

**Figure 4:**
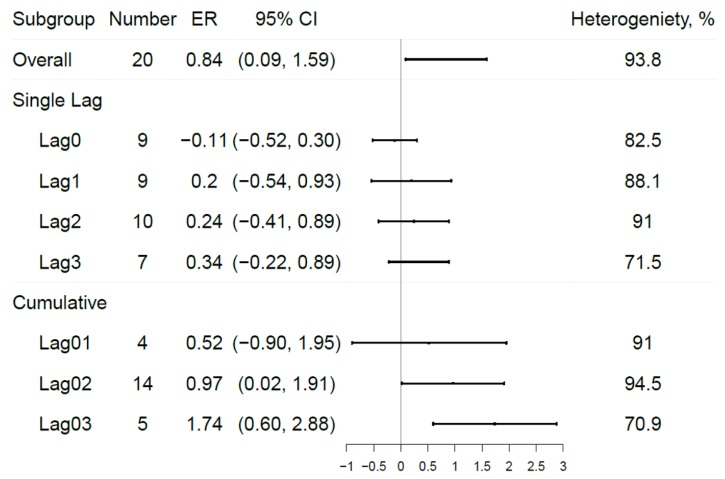
Subgroup analyses among different lag structures. Note: ER, excess risk.

As illustrated by Figure 3, the effect size was weaker among the older population (0.27% 95% CI: −0.02%, 0.57%). This borderline effect, however, lost its significance (0.30% 95% CI: −0.13%, 0.73%) after the estimates from Ding’s study [39] which provided results for different temperature levels, were removed from our analyses. A significant effect was found during the warm but not the cold season, with an increment of 1.06% (95% CI: 0.26%, 1.87%) in COPD hospitalizations per 10 µg/m^3^ increase in maximum 8-h ozone level. As for the different lag structures (Figure 4), the single-day lag results generally showed insignificant and lower pooled results, while the multiple-day lag gained statistical significance with bigger effect estimates during longer day intervals.

## 4. Discussion

These 26 carefully selected publications provided sufficient data for our systematic review and meta-analysis to investigate the relationship between short-term ozone exposure and COPD hospitalizations. Of all the air pollutants, ozone is expected to have the strongest correlation with both temporal and meteorological factors, thus only studies with both variables adjusted were used in our analyses. Considering the confounding effects from age and season, subgroup excess risk was estimated only from all age-range populations and year-round ozone exposure. Our analyses demonstrate that, after short-term exposure, each 10 µg/m^3^ increase in the maximum 8-h ozone concentration was related to a 0.84% (95% CI: 0.09%, 1.59%) increase in COPD hospitalizations. The overall effect size detected in our study is relatively smaller than that in previous meta-analysis, which could be due to our more stringent criteria for effect data extraction.

Meta-regression analyses were used to investigate effect modifiers for overall risk estimates. The analyses show that study designs contributed to borderline significant modifications on health effects from ozone exposure. However, this could be a chance finding, because the differential effects of case crossover design came mainly from two studies. From our analyses, no significant modifying effect was observed for influenza-adjusted status, while Wong et al. [53] found that when the influenza intensity increased from 0% to 10%, the excess risk per 10 µg/m^3^ increase in ozone level increased 0.40% for COPD hospitalizations in the ≥ 65 age group. This heterogeneity could be due to the limited statistical power of the small sample size for our meta-analysis and the relatively low modifying effects. Although insignificant modifying effects were found for different data sources, it should be emphasized that, when examining the association between air pollution exposure and respiratory health effects that require medical attention, it is important to distinguish between hospital admissions and emergency department visits. This is because only a small percentage of respiratory emergency department visitors are admitted to hospitals. Therefore, emergency department visits (especially by self-referrals) may represent less serious but more common outcomes [14].

Results from our subgroup analyses suggest that age and seasonal variations existed. Consistent with findings from previous studies [41,47], stronger associations were found during the warm season, which can be explained by the following: (a) ozone concentrations are typically higher during the warm season; (b) individuals tend to engage in more outdoor activities during the warm season, resulting in even higher exposure levels. As for the relatively weak effects estimated among the old-age group, the result is also consistent with a similar meta-analysis [14] and a multicity study [40]. A previous weight-of-evidence study [21], based on mortality data, concluded that the elderly were at high respiratory health risk from short-term ozone exposure, but the result was inconsistent with morbidity evidence [40]. Unlike asthma or other respiratory diseases, the nature and sites of inflammation among COPD patients are quite different and may be related to different responses to environmental pollutants [54]. Therefore, COPDs may have a different pathology and different clinical manifestations from other respiratory diseases. In addition, symptomatic responses, such as cough or shortness of breath, to ozone exposure tend to increase with age until early adulthood, and then gradually decrease with increasing age [9]. This could influence the effect estimates for the old-age group because they are more tolerant to high ozone pollution.

To our knowledge, this is the first meta-analysis and systematic review which has addressed the differences by lag selection and co-pollutants. Our co-pollutant subgroup analyses included estimates only from the all-age-range populations and from year-round ozone exposure, to avoid further confounders. Although the numbers of estimates were not large, our results demonstrate that short-term ozone exposure was positively associated with COPD hospitalizations, using co-pollutants-adjusted data. Our study also identified meaningful results from different lag subgroups. Many previous studies reported have observations for the lag with the most statistically significant results, instead of all lag structures, but this would bias estimates upward. However, if associations were observed in a multiple-day lag selection but not in a single-day lag selection, single-day lag investigations may underestimate the effects, which is well demonstrated in our results. Indeed, the single-day lag results generally show insignificant and lower pooled results, while the multiple-day lag results show statistical significance with bigger effect estimates during longer day intervals.

Current short-term ambient ozone quality standards for most countries are solely based on daily maximum 8-h exposure concentrations [55]; our results indicate that multiple-day averages should also be considered. However, our ability to analyze trends in even longer time intervals was limited, because most studies did not report results of all lag structures. It is quite likely that the cumulative effects we found were in existence within three days, which is strongly indicated in previous ozone-related mortality studies [47,56]. Our results also indicate that the lag selection protocol might bias the pooled results when performing meta-analyses of ozone-related epidemiological studies. Whether this lag selection bias exists in other air pollution meta-analyses remains unknown.

Several limitations should be considered here. In most of the selected studies, confounding factors were adjusted differently to each other, which might lead to bias. However, as all the selected studies were controlled for the crucial confounders, for example, temperature, relative humidity, day of week, and long-term factors for the time-series, the limitations would not cause a significant impact on our results. Additionally, the subgroup results are based on relatively small numbers of selected studies. More studies are needed to verify our results.

## 5. Conclusions

In summary, our results show that short-term ambient ozone exposure was positively associated with COPD hospitalizations and no publication bias was found. The subgroup analyses indicate that the relationship might vary among different age ranges and seasons. The multiple-day lag trend, with more significant effect estimates during longer day intervals, indicates that a multiple day exposure metric needs to be considered for establishing ambient ozone quality standards. However, this lag trend still needs further investigation. Future environmental studies and meta-analyses, which investigate the health effects of short-term ozone exposure, should also consider a lag selection protocol.

## Figures and Tables

**Table 1:** Contextual details of studies included in the meta-analysis.

Author	Location	Published	Period	Study Design	Data Source	Population	Definition of Outcome	Influenza
Malig et al.	USA	2016	2005–2008	Case crossover	EDVs	All	ICD-9	Adjust
Pothirat et al.	Thailand	2019	2016–2017	Time series	EDVs & HAs	All	ICD-10	No
Szyszkowicz et al.	Canada	2018	2004–2011	Case crossover	EDVs	≥55 years	ICD-10	Adjust
Lee et al.	China	2007	1996–2003	Case crossover	HAs	All	ICD-9	No
Fusco et al.	Italy	2001	1995–1997	Time series	HAs	All	ICD-9	Adjust
Schwartz et al.	USA	1994	1986–1989	Time series	HAs	≥65 years	ICD-9	No
Morgan et al.	Australia	1998	1990–1994	Time series	HAs	≥65 years	ICD-9	No
Tenias et al.	Spain	2002	1994–1995	Time series	EDVs	≥14 years	ICD-9	No
Peel et al.	USA	2005	1993–2000	Time series	EDVs	All	ICD-9	Adjust
Liang et al.	China	2019	2013–2017	Time series	HAs	≥18 years	ICD-10	No
Reid et al.	USA	2019	2008	Time series	EDVs & HAs	All	ICD-9	No
Yang et al.	Canada	2005	1994–1998	Time series	EDVs	≥65 years	ICD-9	No
Halonen et al.	Finland	2010	1998–2004	Time series	EDVs & HAs	≥65 years	ICD-10	Adjust
Anderson et al.	UK	2001	1994–1996	Time series	HAs	≥65 years	ICD-9	Adjust
Qiu et al.	China	2013	1998–2007	Time series	EDVs	All	ICD-9	Adjust
Dab et al.	France	1996	1987–1992	Time series	HAs	All	ICD-9	Adjust
Ko et al.	China	2007	2000–2004	Time series	EDVs	All	ICD-9	No
Hinwood et al.	Australia	2006	1992–1998	Case crossover	HAs	All	ICD-9	No
Arbex et al.	Brazil	2009	2001–2003	Time series	EDVs	≥40 years	ICD-10	No
Ding et al.	China	2017	2000–2013	Case crossover	EDVs	65–79 years	ICD-9	No
Yang et al.	China	2007	1996–2003	Case crossover	HAs	All	ICD-9	No
Schouten et al.	Netherlands	1996	1977–1989	Time series	EDVs	All	ICD-9	Adjust
Strosnider et al.	USA	2018	2000–2014	Time series	EDVs	≥19 years	ICD-9	No
Anderson et al.	Europe	1997	1977–1992	Time series	EDVs	All	ICD-9	Adjust
Stieb et al.	Canada	2009	1992–2003	Time series	EDVs	All	ICD-9/ICD-10	No
Medina-Ramon et al.	USA	2006	1986–1999	Case crossover	HAs	≥65 years	ICD-9	No

Note: EDVs, emergency department visits; HAs, hospital admission visits.

## Supplementary Materials

The following are available online at https://www.mdpi.com/1660-4601/17/6/2130/s1, Table S1: Summary of former meta-analysis articles about short-term ambient level ozone exposure and COPD hospitalizations, Table S2: Summary of study location, study period, ICD codes and weather, Table S3: Summary of adjusting confounders and statistical models, Figure S1: Influence analyses, Figure S2: Forest plot with influential case removed, Figure S3: Leave-one-out analyses with influential case removed, Figure S4: Funnel plot for meta-analysis, Figure S5: Funnel plot with influential case removed, Figure S6: Meta-regression analyses for the modifying effect of different study designs, Figure S7: Meta regression analyses with influential case removed.
