# Supplementary material for: A Systematic Review and Meta-Analysis of Short-Term Ambient Ozone Exposure and COPD Hospitalizations
Source: Int J Environ Res Public Health. 2020 Mar 23;17(6):2130. doi: 10.3390/ijerph17062130 (PMC7143242; doi:10.3390/ijerph17062130)

e-Table 1. Summary of former meta-analysis articles about short-term ambient level ozone exposure and COPD hospitalizations.

Author	Searching time	Database	COPD Definition	Adjusted factors
Ji et al, 2011	1990 - 2008	PubMed	ICD-9, ICD-10, APR-DRG	age, season, lag, region, disease definition, type of hospital visits, ozone metric
Zhang et al, 2016	- December 2014	PubMed, Web of Science	NA	age, type of hospital visits
Li et al, 2016	- March 30, 2016	Web of Science, Ovid, Embase, Environmental Science and Pollution Management Index, CINAHL, Google Scholar, Cochrane, CNKI	ICD-10, Lung function test	age, lag, study design, study location
Moore et al, 2016	1980 - September 2015	MEDLINE, Embase, BIOSIS, Science Citation Index	ICD-9, ICD-10	season, study location

e-Table 2. Summary of study location, study period, ICD codes and weather.

Author	Study location	Period	Definition of outcome	8-hour ozone (μg/m^3)	Temperature (℃)
Malig et al	California, USA	2005 - 2008	ICD-9: 490-492, 494-496	48.5 - 80.9	8.2 - 18.8
Pothirat et al	Chiang Dao district, Chiang Mai, Thailand	March 1, 2016 - March 31, 2017	ICD-10: J44.1	75.7 (54.7, 128.6)	20.35 (14.58, 34.28)
Szyszkowicz et al	Ontario, Canada	April, 2004 - December, 2011	ICD-10: J41-J44	3.7 - 294	-26.6 - 32.0
Lee et al	Kaohsiung, Taiwan	1996 - 2003	ICD-9: 490-492, 494, 496	94.1 (55.1, 129.8)	26.3 (22.6, 28.2)
Fusco et al	Rome, Italy	January 1, 1995 - October 31, 1997	ICD-9: 490-492, 494-496	24.0 (13.3, 37.2)	16.8 (11.6, 22.3)
Schwartz et al	Detroit, Michigan, USA	1986 - 1989	ICD-9: 491,492, 494-496	69.8 (44.1, 102.9)	10 (1.7, 19.4)
Morgan et al	Sydney, Australia	January, 1990 - December, 1994	ICD-9: 490-492, 494, 496	32.3 (26.5, 42.6)	18 (14, 21)
Tenias et al	Valencia, Spain	January 1, 1994 - December 31, 1995	ICD-9, excluding asthma	10 - 118	6.6 - 33.8
Peel et al	Atlanta, USA	January 1, 1993 - August 31, 2000	ICD-9: 491, 492, 496	109 ± 46.6	17.5 ± 8.3
Liang et al	Beijing, China	January 18, 2013 - December 31, 2017	ICD-10: J44.0 - J44.9	83 (50, 135)	14 (2, 23)
Reid et al	California, USA	May 6, 2008 - September 26, 2008	ICD-9: 491, 492, 496	NA	NA
Yang et al	Vancouver, British Columbia, Canada	1994 - 1998	ICD-9: 490-492, 494, 496	3.7 - 141.9	-9.2 - 25.9
Halonen et al	Helsinki, Finland	1998 - 2004	ICD-10: J41, J44	71.3 (58.6, 84.1)	14.3 (11.5, 17.2)
Anderson et al	West Midlands conurbation, UK	October 1994 - December 1996	ICD-9: 490-492, 494-496	0.8 - 176.2	-3.4 - 22.6
Qiu et al	Hong Kong, China	January 1, 1998 - December 31, 2007	ICD-9: 491, 492, 496	39.8 ± 24.3	23.6 ± 4.9
Dab et al	Paris, France	January 1, 1987 - September 30, 1992	ICD-9: 490-492, 494-496	36 (6, 147)	NA
Ko et al	Hong Kong, China	January, 2000 - December, 2004	ICD-9: 491, 492, 496	26.7 (18.1, 41)	25.2 (19.9, 27.9)
Hinwood et al	Perth, Australia	1992 - 1998	ICD-9: 490.00 - 496.99, excluding asthma	50.8 ± 12.7	18.4 ± 4.5

Arbex et al	Sao Paulo, Brazil	February 1, 2001 - December 31, 2003	ICD-10: J40-J44	166.2 (119.7, 224.4)	15.8 (13.1, 18.2)
Ding et al	Taipei, Taiwan	January 2000 - December 2013	ICD-9: 491, 492, 496	99.9 (81.4, 122.9)	23.8(19.2, 27.9)
Yang et al	Taipei, Taiwan	1996 - 2003	ICD-9: 490-492, 494, 496	72.6 (52.4, 93.9)	23.8 (19.2, 27.6)
Schouten et al	Amsterdam, Rotterdam, Netherlands	1977 - 1989	ICD-9: 490-492, 494, 496	Amsterdam: 69 (5, 134); Rotterdam: 61 (6, 140)	10.1 (-1.4, 19.1)
Strosnider et al	17 states, USA	2000 - 2014	ICD-9: 491, 492, 496	NA	NA
Anderson et al	Six European cities (Amsterdam, Barcelona, London, Milan, Paris, Rotterdam)	1977 - 1992	ICD-9: 490, 491, 492, 496	NA	NA
Stieb et al	Seven Canadian cities (Montreal, Ottawa, Edmonton, Saint John, Halifax, Toronto, Vancouver)	1992 - 2003	ICD-9: 490-492, 494-496; ICD-10: J40-J44, J47, J67	NA	NA
Medina-Ramon et al	36 cities, USA	1986 - 1999	ICD-9: 490-492, 494-496	NA	NA

e-Table 3. Summary of adjusting confounders and statistical models.

Author	Confounders	statistical methods
Malig et al	Apparent temperature	Conditional logistic regression
Pothirat et al	Temperature, wind speed, pressure, rainfall, relative humidity	Generalized linear Poisson model
Szyszkowicz et al	Temperature, relative humidity	Conditional logistic regression
Lee et al	Temperature, relative humidity	Conditional logistic regression
Fusco et al	Temperature, mean humidity, day of study, day of the week, holidays	Generalized additive Poisson model
Schwartz et al	Temperature, dew point temperature, long-term trend, seasonal trend, month	Generalized additive Poisson model
Morgan et al	Temperature, dew point temperature, seasonal trend, day of the week, holidays	Generalized additive Poisson model
Tenias et al	Temperature, relative humidity, seasonal trend, long-term trend, day of the week, feast days	Autoregression poisson model
Peel et al	Temperature, dew point temperature, long-term trend, day of the week, holiday, daily pollen counts	Generalized additive Poisson model
Liang et al	Temperature, relative humidity, long-term trend, seasonal trend, day of the week, holiday	Generalized additive Poisson model
Reid et al	Daily heat index, temporal trend, age, race, income, day of week, holiday	Generalized additive Poisson model
Yang et al	Day of the week, temporal trends, annual trends	Generalized additive Poisson model
Halonen et al	Temperature, relative humidity, long term trend, barometric pressure, high pollen episodes, holiday	Generalized additive Poisson model
Anderson et al	Temperature, relative humidity, seasonal trend, day of the week, holiday	Generalized additive quasi-likelihood model
Qiu et al	Temperature, relative humidity, seasonal trend, day of the week, holiday	Generalized additive Poisson model
Dab et al	Temperature, relative humidity, long term trend, seasonal, weekly and daily patterns, holiday	Autoregression poisson model
Ko et al	Temperature, relative humidity, time trend, day of the week, holiday	Generalized additive Poisson model
Hinwood et al	Temperature, maximum humidity, day of the week, holiday	Conditional logistic regression
Arbex et al	Minimum temperature, humidity, long term trends, seasonal trend, day of the week	Generalized linear Poisson model
Ding et al	Temperature, relative humidity, air pressure difference	Conditional logistic regression
Yang et al	Temperature, relative humidity	Conditional logistic regression
Schouten et al	Temperature, relative humidity, year, day of the week, holiday	Autoregression poisson model
Strosnider et al	Temperature, dew point temperature, calendar date, day of the week, holiday	Bayesian hierarchical model

Anderson et al	Temperature, relative humidity, long term trend, seasonal, day of the week, holiday	Poisson time series regression
Stieb et al	Temperature, relative humidity, time of day, seasonal, day of the week, holiday	Generalized linear quasi-Poisson model
Medina-Ramon et al	Temperature, day of the week	Conditional logistic regression model

e-Figure 1. Influence analyses.

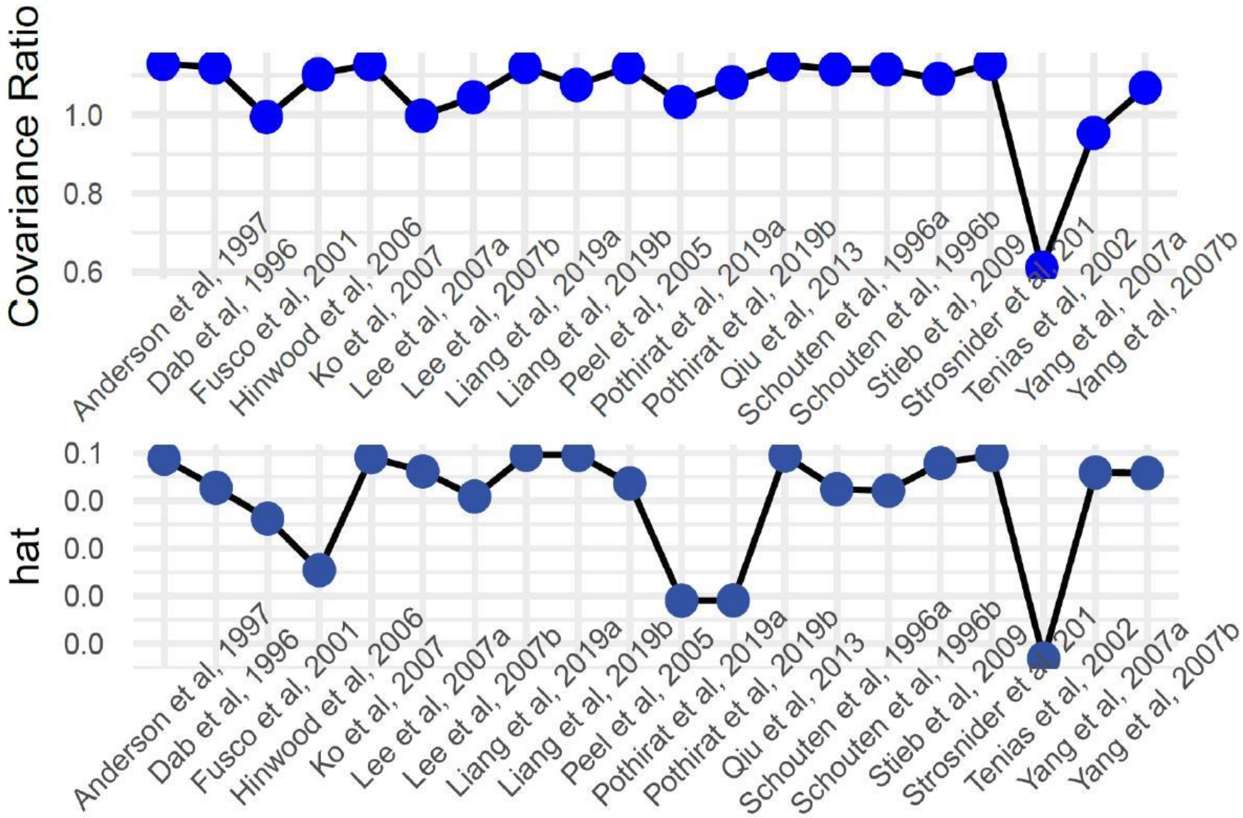

e-Figure 2. Forest plot with influential case removed.

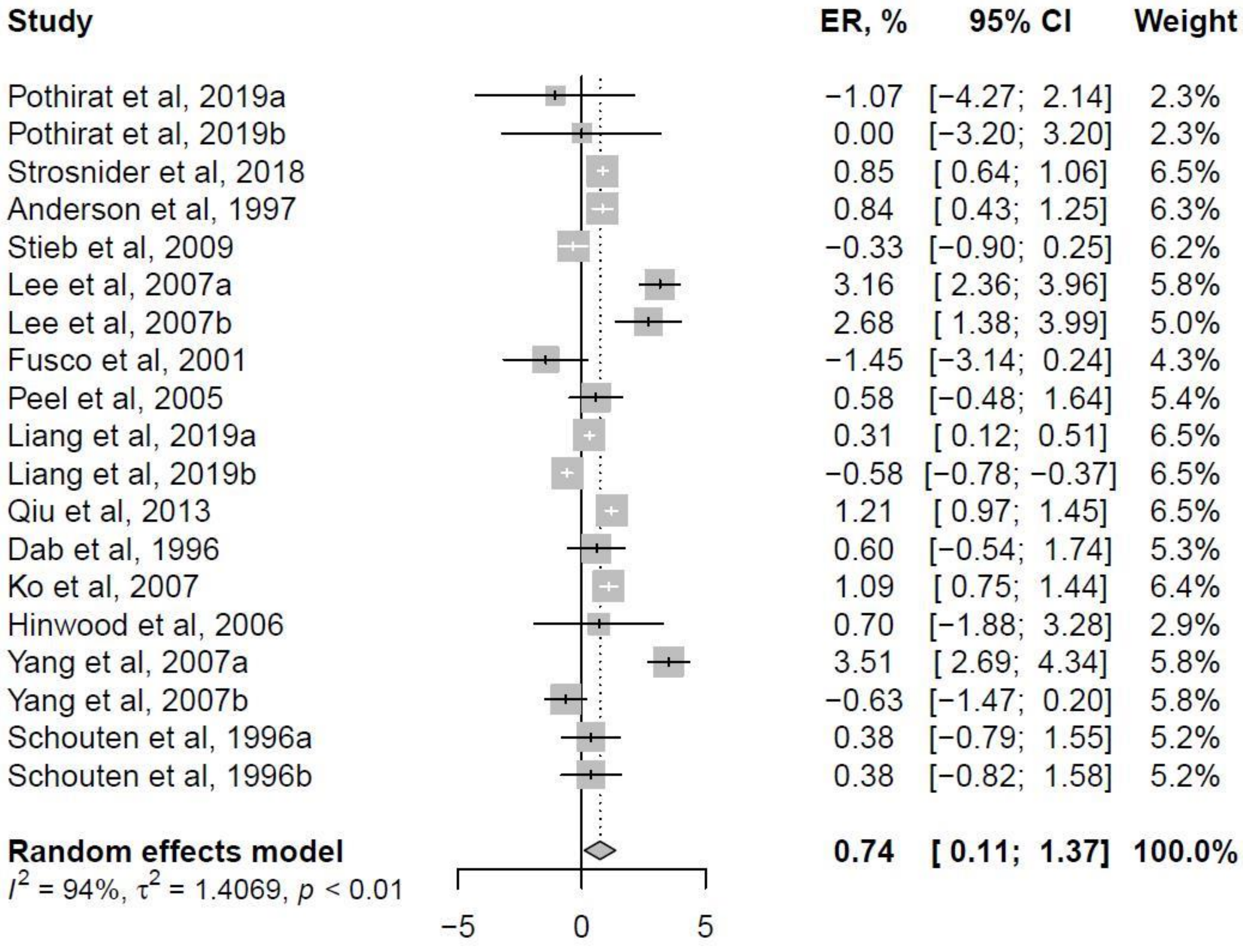

e-Figure 3. Leave-one-out analyses with influential case removed.

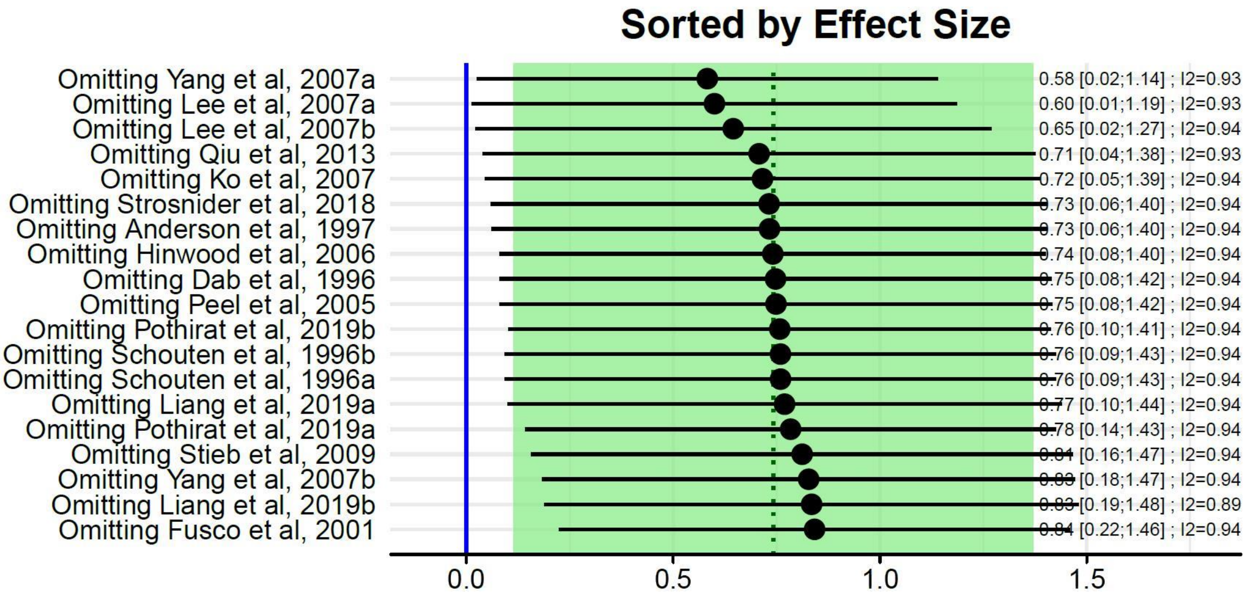

e-Figure 4. Funnel plot for meta-analysis.

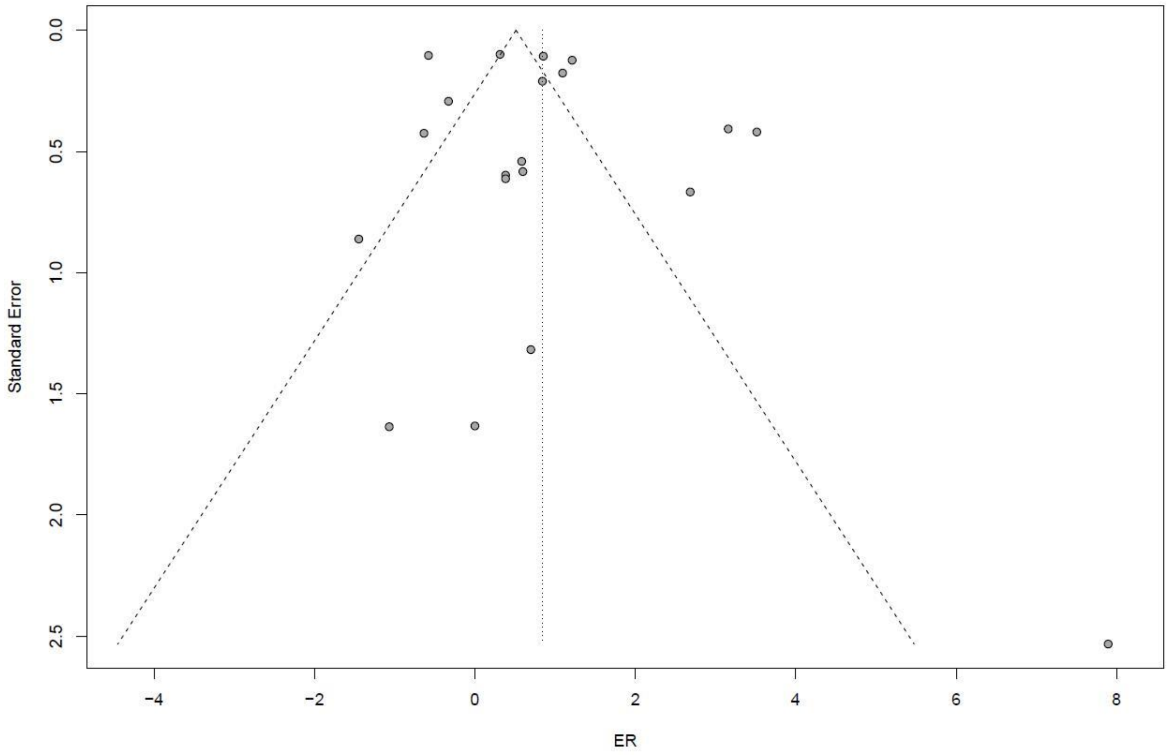

e-Figure 5. Funnel plot with influential case removed.

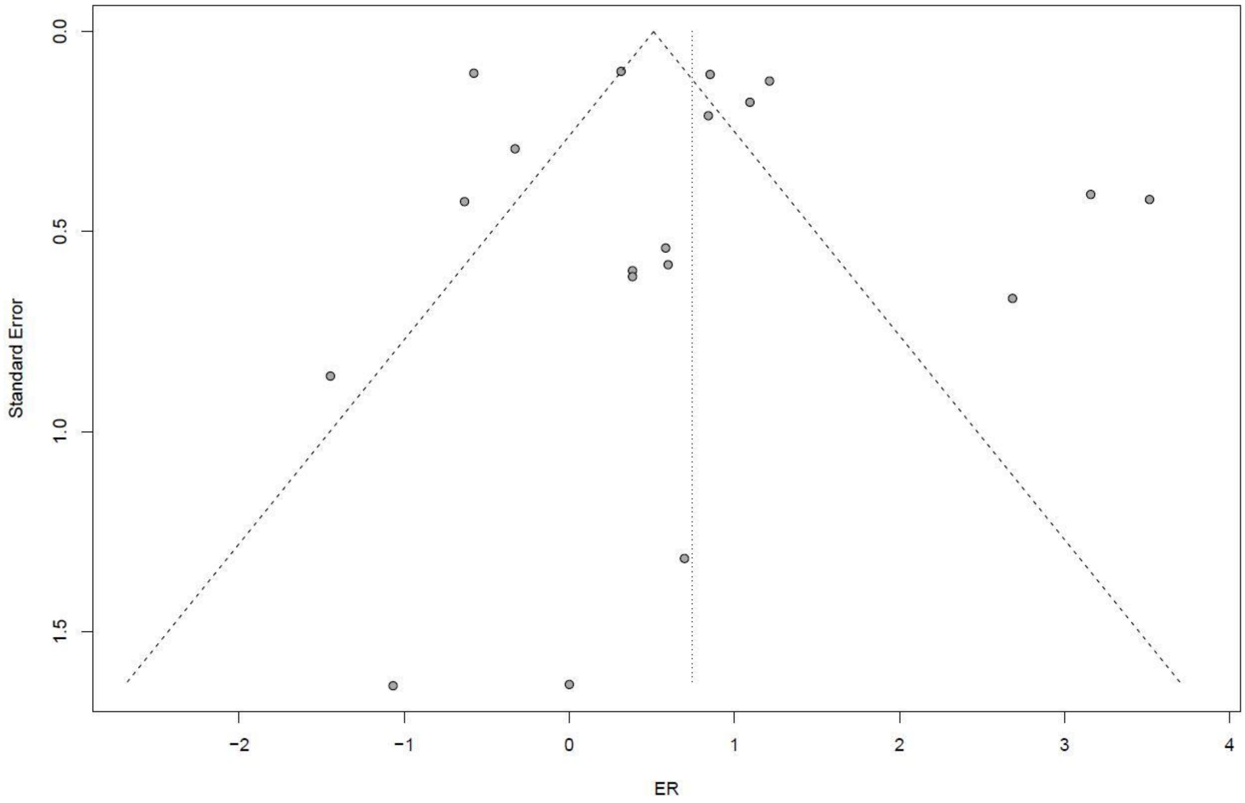

e-Figure 6. Meta-regression analyses for the modifying effect of different study designs.

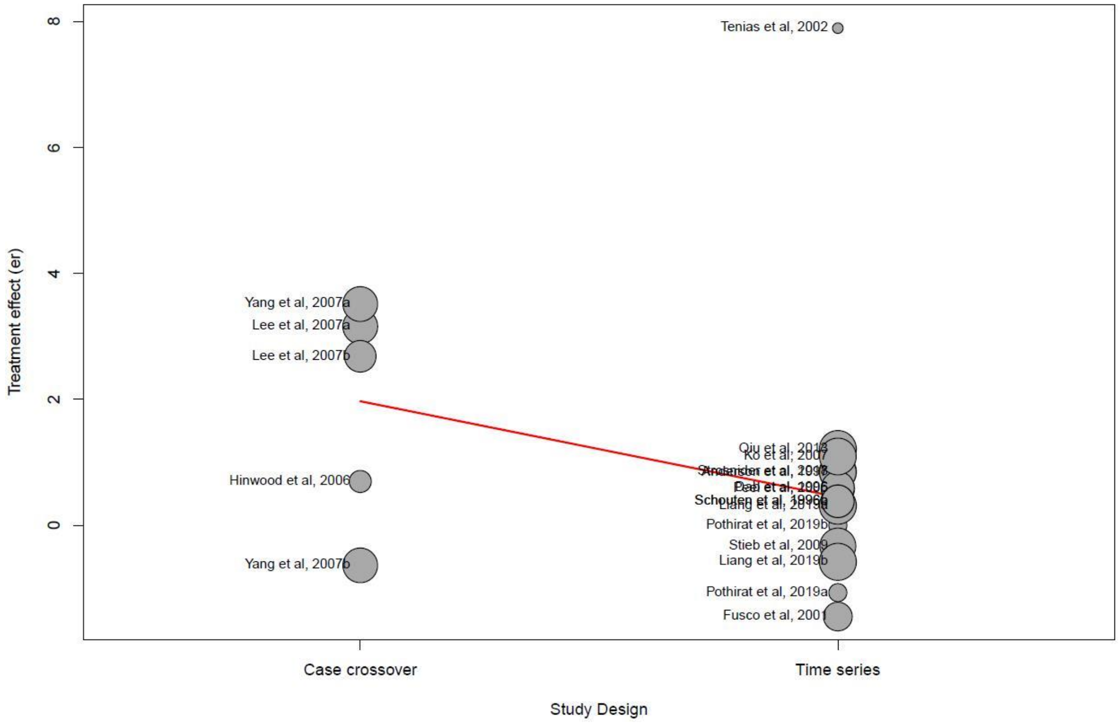

e-Figure 7. Meta regression analyses with influential case removed.

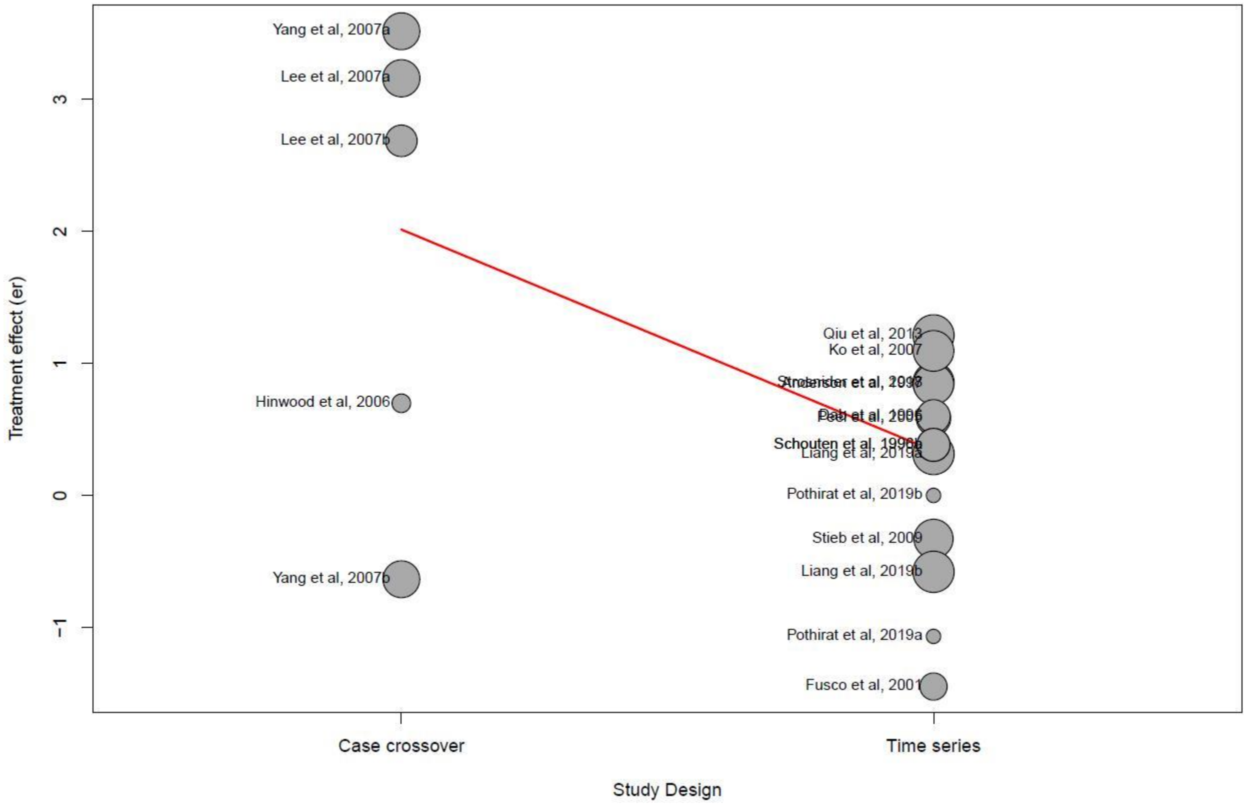

Supplement: Supplementary file 1 [file ijerph-17-02130-s001.pdf]
